# Extracellular stressors change BBSome expression of benign mesothelial and primary pleural mesothelioma cells and affect cell adhesion and migration

**DOI:** 10.14814/phy2.70983

**Published:** 2026-06-16

**Authors:** Rajesh M. Jagirdar, Erasmia Rouka, Eleanna Pitaraki, Sotirios I. Sinis, Charalambos Varsamas, Eleftherios D. Papazoglou, Lydia Giannakou, Panagiotis I. Tzamalas, Ourania S. Kotsiou, Anastasios Giannou, Chrissi Hatzoglou, Najib M. Rahman, Konstantinos I. Gourgoulianis, Sotirios G. Zarogiannis

**Affiliations:** ^1^ Department of Physiology, Faculty of Medicine, School of Health Sciences University of Thessaly Larissa Greece; ^2^ Department of Nursing, School of Health Sciences University of Thessaly Larissa Greece; ^3^ Department of Respiratory Medicine, Faculty of Medicine, School of Health Sciences University of Thessaly Larissa Greece; ^4^ Section of Molecular Immunology and Gastroenterology, I. Department of Medicine UKE Hamburg Germany; ^5^ Department of General, Visceral and Thoracic Surgery University Medical Center Hamburg‐Eppendorf Hamburg Germany; ^6^ Hamburg Center for Translational Immunology (HCTI), University Medical Center Hamburg‐Eppendorf Hamburg Germany; ^7^ University of Oxford Respiratory Trials Unit, Churchill Hospital Oxford UK; ^8^ NIHR Oxford Biomedical Research Centre University of Oxford Oxford UK

**Keywords:** BBSome, hyperosmotic stress, malignant pleural mesothelioma, oxidative stress, primary cilium

## Abstract

Pleural mesothelial cells have a primary cilium (PC), a solitary sensory organelle facing the extracellular environment. The BBSome is critical for the PC function and comprises several BBS proteins. Extracellular stimuli, like the ones encountered during a pleural effusion (osmotic, inflammatory, and oxidative stress‐related signals) could influence the PC and BBSome. Using 2D and 3D culture models of benign mesothelial and primary malignant pleural mesothelioma cells we explored the BBSome components gene expression under hyperosmotic, inflammatory, and oxidative stress. We also assessed their effects in combination with PC perturbing drugs in the context of cell adhesion and cell migration, which are critical for tissue healing. These extracellular stimuli changed the expression patterns of *BBS* genes, while changes in cell adhesion were dependent on the cell and stimulus type. Cell migration was also sensitive to stress stimuli and the native PC length was critical in this context. Our results provide considerable insight into the molecular and phenotypical changes underlying PC responses of benign and malignant mesothelial cells to extracellular stimuli. Further research will be needed to assess the potential therapeutic implications of PC extracellular stimulation in malignant pleural disease.

## 1 INTRODUCTION

The primary cilium (PC) is a sensory organelle receiving extracellular cues and propagating them within the cell (Mill et al., 2023). A significant molecular component of PC is the BBSome, comprising proteins encoded from a set of genes that are deregulated in the Bardet‐Biedl Syndrome, a genetic ciliopathy (Tian et al., 2023). Mesothelial cells possess PC (Bird, 2004; Bird et al., 2004) and pleural mesothelial cells (PMCs) have been described to possess PC in both benign and malignant states (Barbarino et al., 2022; Rouka et al., 2023). The PC contributes to mesothelial cell viability, migration, tumor spheroid formation, and collagen extracellular matrix invasion, as well as in the mesenchymal potential and variably in cell adhesion, highlighting its seminal role in mesothelial physiology (Jagirdar et al., 2023). The above was demonstrated using PC‐modulating drugs that either cause PC shedding (or autotomy) or PC elongation (Jagirdar et al., 2023). Additionally, the PC‐associated BBSome components were differentially expressed when cells were cultured in 2D or 3D cultures (Rouka et al., 2023).

The extracellular environment for pleural mesothelial cells varies in health and disease, since the nature of the pleural effusion depends on the underlying pathology (Peppa et al., 2021; Zarogiannis, Tsilioni, et al., 2013). Pleural effusion fluid can have high indices of oxidative stress (Tsilioni et al., 2011; Vavougios et al., 2015), inflammatory stress (Hou et al., 2022), and differential protein levels and osmolality (Katkova et al., 2019; Peppa et al., 2021; Zarogiannis, Tsilioni, et al., 2013). Malignant pleural effusion fluid cytology has demonstrated the presence of single cells as well as 3D clusters of cells in multiple types of malignancies (Jagirdar et al., 2021; Papazoglou et al., 2019; Surina et al., 2023).

The potential PC‐associated changes in pleural mesothelial cells during the effect of extracellular stimuli relevant to pleural effusions is unknown. Here we assessed the gene expression of *BBS* genes in the human benign mesothelial cell line MeT‐5A, and in primary malignant pleural mesothelioma (pMPM) cells during hyperosmotic, oxidative, and inflammatory stress. Under these stressors, the role of innate PC maintenance was also assessed on cell adhesion and cell migration.

## 2 MATERIALS AND METHODS

### 2.1 Cell culture

The human cell line MeT‐5A (benign immortalized mesothelial cells, male donor), and primary epithelioid MPM cells from a male patient were used. Cells were cultured with 10% Fetal Bovine Serum‐RPMI (F0804, Sigma, St Louis, MO, USA), 2 mM L‐Glutamine (G7513, Sigma), 1% Penicillin/Streptomycin (P4333, Sigma), and 0.5% w/v Plasmocin (ANT‐MPP, InvivoGen, Toulouse, France) in a 5% CO_2_ incubator. Cells were synchronized by serum starvation in 0.5% FBS‐RPMI over 24 h before experiments. Cells were cultured in 2D at a density of 1.5 × 10^6^ cells in 3 mL volumes on 60 mm petri dish surfaces (REF 430166, Corning, USA) pretreated with bovine plasma fibronectin (FN) (F4759‐1MG, Sigma) or as 3D spheroids (10^5^/25 μL) in a hanging drop model on sterile microbiology plate lids (P5981, Sigma) with 250 ng/mL FN as described previously (Jagirdar et al., 2021). The isolation process of the pMPM cells from the pleural effusion of the male patient with epithelioid MPM has been described previously (Jagirdar et al., 2023). The patient provided written informed consent, and the study was conducted according to the Declaration of Helsinki and was approved by the Ethics Committee of the University Hospital of Larissa (29268/16‐07‐2019).

### 2.2 qPCR assay

Primers specific to human BBSome transcripts were designed with the NCBI Primer‐BLAST tool (Table 1). Quantitative real‐time PCR was performed using b‐actin as the reference gene. Total RNA was extracted using the TRIzol™ Reagent (Invitrogen™) and quality was assessed with a NanoDrop spectrophotometer (Thermo Fisher Scientific). 200 ng of RNA were reverse transcribed into cDNA in a 20 μL reaction volume using the SuperScript™ III first‐strand synthesis system (Catalog # 18080051, Invitrogen™). cDNA was diluted 1:5 with nuclease‐free water (W4502‐1 L, Sigma‐Aldrich®). Amplifications of diluted cDNA were performed using the PowerUp™ SYBR™ Green Master Mix (A25742, Applied Biosystems™) and 0.4 μM primers on the ABI 7300 Real‐Time PCR System. Thermocycler conditions were: 50°C for 2 min, 95°C for 2 min followed by 40 cycles of 95°C for 15 s, 55°C for 30 s, and 72°C for 1 min. Changes to BBSome gene expression was done by ΔΔCt method. ΔCt values were converted to 2^−ΔΔCt^ to assess the fold change followed by log_2_ transformation. All experiments were performed 3 times in duplicates.

**TABLE 1:** Primer pair characteristics of BBSome genes.

Gene (symbols)	Primer sequences (5′‐>3′)	Length of product	Template
*BBS1*	Forward: TTTAGCCAAGATGAGCCTTCC Reverse: TGCAGTACTTGGGGTGCTTG	143	NM_024649.5
*BBS2*	Forward: AAACTGCGCCACAAAATCAGC Reverse: GGATTATGAATAAAAACCTTGCCCG	110	NM_001377456.1
*BBS4*	Forward: ATTGGCCTAGGAGATCAGCC Reverse: CAACTGGTCTTGTGCCTTGT	79	NM_001252678.1
*BBS5*	Forward: CTTGTTCACCATGTCGGTGC Reverse: GGTCTTGTTTTCATTTGCTGCG	87	NM_152384.3
*BBS7*	Forward: AGAGGCAGATCACCTACAGGA Reverse: ATCAGTGATCATGCCATAGAGT	76	NM_176824.3
*BBS9*	Forward: TTATTCCAGACCAACAGGCAT Reverse: GCAGTTTTTGAAGGCTGACCT	96	NM_001033605.1
*BBS18*	Forward: GGGATGCATCTTTTCTTCTGTATGC Reverse: CTGCAGCTTTAAGCATCCAACC	83	NM_001195305.3

### 2.3 Drug treatments

Cells were serum‐starved in 0.5% FBS‐RPMI media for 24 h before experiments. RPMI‐based culture conditions have been employed in our previous research, including the establishment and characterization of the primary pMPM model used in the current work (Jagirdar et al., 2021, 2023; Papazoglou et al., 2019; Rouka et al., 2023). In the 3D culture model, spheroids were pre‐formed, media was removed with a sterile filter paper, and then the appropriate treatment media was placed for the experimental procedure. Hyperosmotic treatment consisted of 7.2% bovine serum albumin (BSA) (A2153, Sigma), inflammatory stimulus consisted of LPS 5 μg/mL (L4516, Sigma), and oxidative stress using 100 μM H_2_O_2_ (HIO1351000, Scharlau). For deciliation, 30 mM Ammonium Sulfate (AS: A4915‐500G, Sigma), and for ciliary extension, 50 mM Lithium Chloride (LC: L4408‐100G, Sigma) were included in modified media. Drug concentrations were selected based on previously published studies that employed these agents in mesothelial and/or ciliary‐related experiments (Horiuchi et al., 2009; Jagirdar et al., 2023; Rouka et al., 2023; Zhang et al., 2016). LPS, the major component of the outer membrane of gram‐negative bacteria (Zhang et al., 2016), was used as an inflammatory stimulus because infectious pleural diseases usually involve exposure to microbial components.

### 2.4 Cell adhesion assay

For the cell adhesion assay, we used cells that were synchronized by 24‐h serum starvation in 60 mm plates before each experiment. Cell adhesion was performed in FN‐treated 96‐well plates (Cellstar, Cat number 655180). In each well, 2 × 10^4^ cells were seeded in 100 μL 10% FBS‐RPMI media alone or with BSA, LPS, H_2_O_2_ without or with AS or LC. Cells were allowed to attach for 90 min in the incubator, and unattached cells were aspirated by three warm PBS washes. Attached cells were fixed with 4% paraformaldehyde (PFA) followed by 0.5% crystal violet staining for 10 min. The stain was then aspirated and plates were washed in running tap water. Cells were de‐stained with 10% acetic acid, and optical density (OD) measurements at 595 nm were performed. OD values were compared as percentage adhesion compared to the control condition.

### 2.5 Cell migration assay

Assays were carried out in 48 (Corning, 3548) or 96‐well plates pretreated with FN. Cells were cultured to confluency in 10% RPMI and serum starved for 24 h. The monolayer was scratched with a 500‐micron 3D printed plastic tip, washed with warm PBS and then with media consisting of BSA, LPS, or H_2_O_2_ alone or with added AS or LC. Cells were imaged at ×100 magnification with a 40× objective and 10X eye piece on an Olympus TS100 microscope, with an Opticam camera system and Toupview version 3.7 software. The plates were then incubated for 6 h and imaged again. The cell‐free area was measured in ImageJ using the polygon tool. The migration index (MI) was used: MI = (A0 − A6)/A0. A0 represents the area measured at time 0, and A6 represents the area at the time of experiment termination. The 6‐h assay incubation period post serum‐starvation precludes any proliferation‐associated effects.

### 2.6 Statistical analyses

All experiments were performed at least three times. Analyses were performed using Prism 9.0 for Mac (San Diego, CA, USA). The normality of data was assessed by the D'Agostino & Pearson normality test. Data comparisons were performed with one‐way ANOVA for parametric data or the Kruskal–Wallis test for non‐parametric data. Two‐way ANOVA was performed during analyses of experiments with AS or LC during BSA, LPS, or H_2_O_2_ stimulus. All data are presented as mean ± SEM. Values of *p* < 0.05 were deemed significant.

## 3 RESULTS

### 3.1 BBSome genes in 2D culture are differentially affected depending on the cell type under the influence of hyperosmotic, inflammatory, and oxidative extracellular stimuli

**FIGURE 1:**
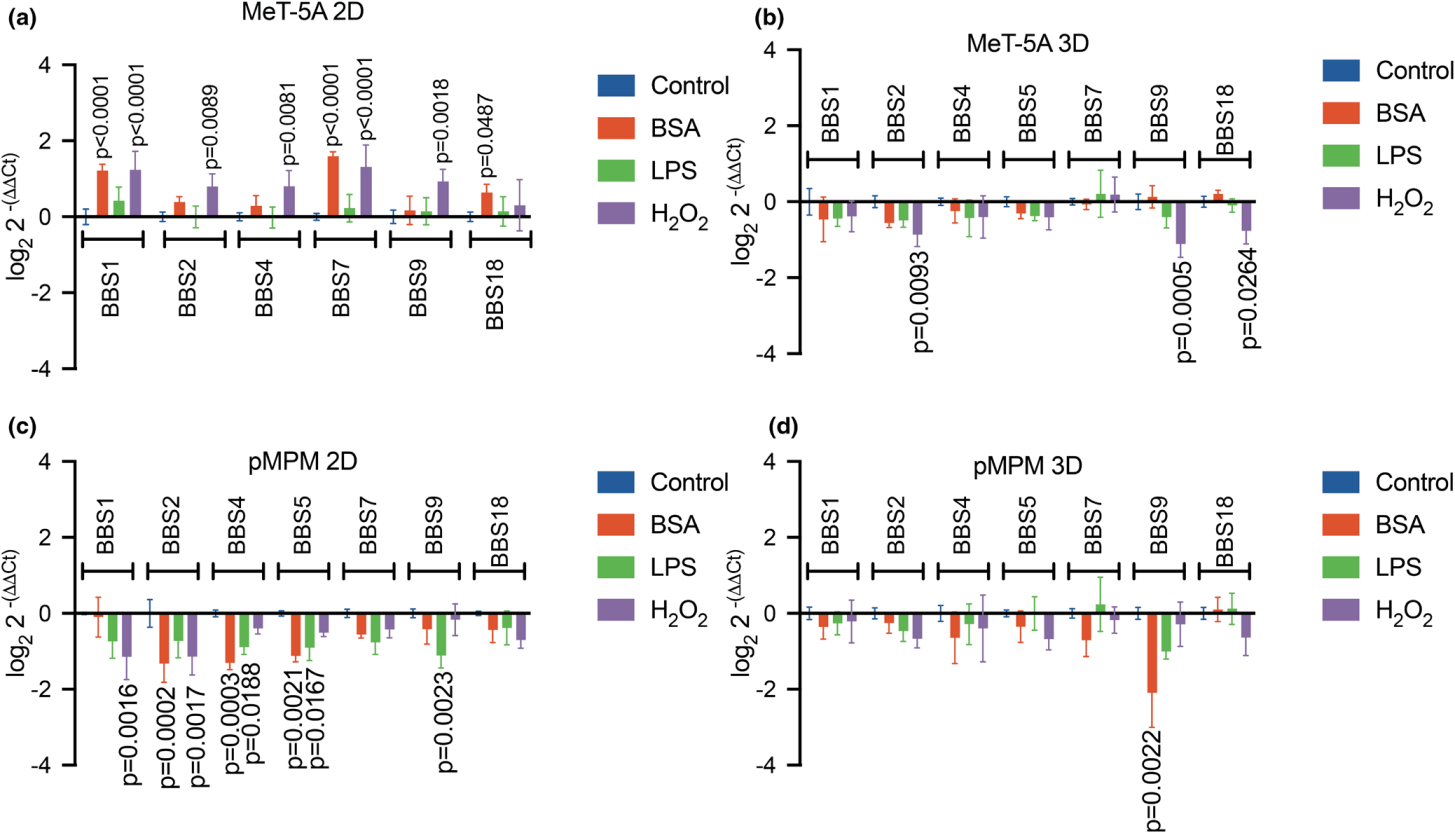
The effect of BSA, LPS, or H_2_O_2_ cell exposure on BBSome genes in MeT‐5A and pMPM cells during 2D and 3D culture conditions. Mean log2 of 2‐ΔΔCt ± SEM with BSA, LPS, or H_2_O_2_. (a) MeT‐5A in 2D, (b) MeT‐5A in 3D, (c) pMPM in 2D, and (d) pMPM in 3D. Actual *p* values are shown vs. untreated controls. Graphed data consist of 3 separate experiments combined, *n* = 6.

We assessed the gene expression of *BBS1*, *BBS2*, *BBS4*, *BBS7*, *BBS8*, *BBS9*, and *BBS18* in 2D cultures of MeT‐5A and the gene expression of *BBS1*, *BBS2*, *BBS4*, *BBS5*, *BBS7*, *BBS8*, *BBS9*, and *BBS18* in pMPM cells. The stimulus that was more potent in inducing significant increases in the gene expression of all tested *BBS* genes in MeT‐5A cells (*p* < 0.01 for *BBS2*, *BBS4*, *BBS9*, and *p* < 0.001 for *BBS1, BBS7* versus Control in all cases), except *BBS18*, was H_2_O_2_. High BSA increased the gene expression of *BBS1*, *BBS7*, and *BBS18* (*p* < 0.001, *p* < 0.001, and *p* < 0.01 respectively versus Control). Interestingly, LPS exposure did not induce any changes in the expression of *BBS* genes (Figure 1a).

In pMPM cells, however, we observed a significant reduction of gene expression in all cases. More specifically, H_2_O_2_ induced a significant decrease in *BBS1* and *BBS2* expression (*p* < 0.001 in both cases versus Control). High BSA significantly reduced the expression of *BBS2*, *BBS4*, and *BBS5* (*p* < 0.001, *p* < 0.001, and *p* < 0.01 respectively vs. Control). Lastly, LPS also induced a significant reduction of *BBS4*, *BBS5*, and *BBS9* expression levels (*p* < 0.05, *p* < 0.05, and *p* < 0.01, respectively vs. Control) (Figure 1b).

### 3.2 BBSome gene expression in 3D cultures of both MeT‐5A and pMPM is much less influenced by the studied extracellular stimuli

In 3D cultures, the effects of the extracellular stimuli were much less pronounced. Indeed, in MeT‐5A spheroids only the H_2_O_2_ induced significant changes that involved the reduction in the gene expression of *BBS2*, *BBS9*, and *BBS18* (*p* < 0.01, *p* < 0.001 and *p* < 0.05 respectively vs. Control; Figure 1c). High BSA and LPS did not elicit any effects *on BBSome* transcripts. Finally, in 3D cultures of pMPM, neither H_2_O_2_ nor LPS had any effect on the *BBSome* gene expression in both MeT‐5A and pMPM cells. High BSA on the other hand reduced significantly the gene expression of *BBS9* (*p* < 0.01 vs. Control; Figure 1d).

### 3.3 Osmotic, inflammatory, and oxidative stress induce a variable effect on the cell adhesion of MeT‐5A and pMPM cells

**FIGURE 2:**
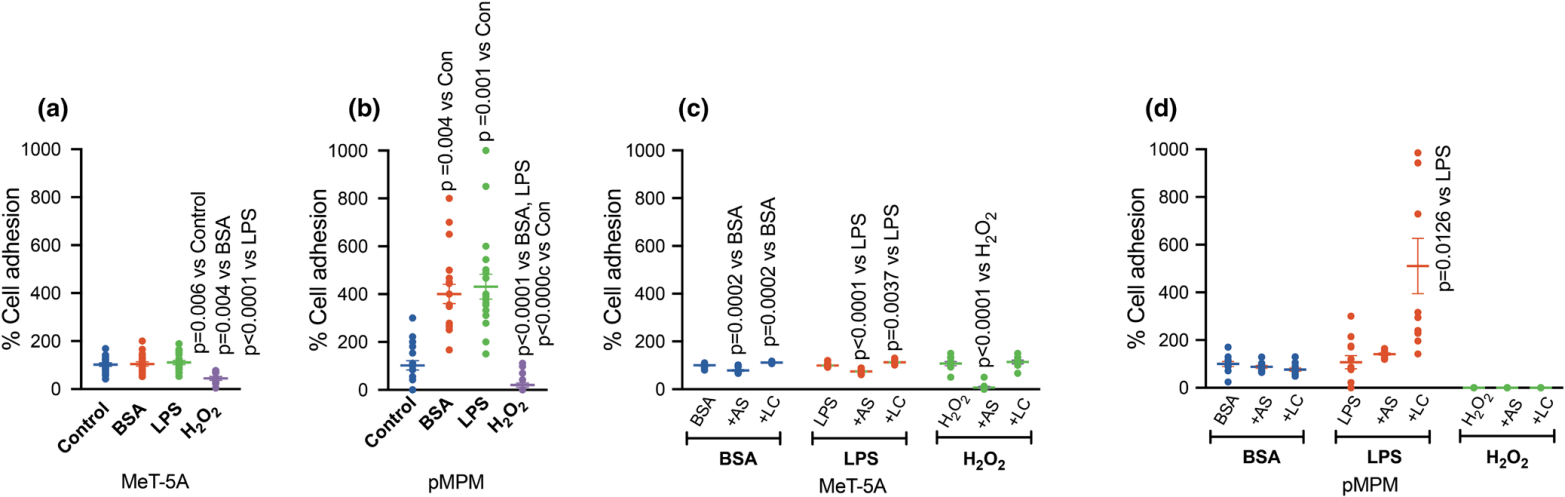
The effect of BSA, LPS, or H_2_O_2_ exposure during cell adhesion. Mean values of cell adhesion ± SEM with BSA, LPS, or H_2_O_2_ with treatments (AS, LC) expressed as % of controls. (a, *n* = 18, 3 experiments; c, *n* = 10–12, 3 experiments) MeT‐5A, (b, *n* = 18, 3 experiments; d, *n* = 10–12, 3 experiments) pMPM cells. Actual *p*‐values are shown versus untreated controls or stated for comparisons among the groups.

In MeT‐5A cells, H_2_O_2_ treatment resulted in significantly decreased cell adhesion as compared to control or BSA or LPS (Control 102.1% ± 7.72%, BSA 104.4% ± 9.28%, LPS 111.00% ± 8.98%, H_2_O_2_ 44.28% ± 8.31%; *p* < 0.001 *n* = 10–18; Figure 2a). In pMPM cells, the cell adhesion was significantly increased by BSA (400.7% ± 40.63%, *p* < 0.005) and LPS treatments (431.2% ± 51.9%, *p* < 0.001) compared to controls (Control; 102.5% ± 19.84%, *n* = 18), and conversely significantly reduced by treatment with H_2_O_2_ (20.16% ± 8.68%; *p* < 0.001; Figure 2b).

Next, we determined the role of PC itself during cell adhesion with AS or LC. In MeT‐5A, BSA + AS treatment significantly reduced cell adhesion (78.78% ± 3.50%, *p* < 0.001, *n* = 12) and BSA + LC significantly increased cell adhesion (112.07% ± 0.99%, *p* < 0.01, *n* = 12) compared to BSA control cell adhesion (100.0% ± 2.55%, *n* = 12). During LPS treatment, LPS + AS stimulus significantly reduced cell adhesion while LPS + LC enhanced cell adhesion (LPS Control 100.0% ± 2.28%; LPS + AS 74.76% ± 2.34%; *p* < 0.001, LPS + LC 112.90% ± 2.84%; *p* < 0.01, *n* = 12). In Met5A, cell adhesion was significantly lowered with H_2_O_2_ + AS treatment (H_2_O_2_ Control 107.66% ± 8.84%; +AS 7.00% ± 4.98%; *p* < 0.001, *n* = 10) but not with H_2_O_2_ + LC (Figure 2c). In pMPM cells, under BSA + AS and BSA + LC conditions, cell adhesion was unperturbed compared to BSA control cell adhesion. During LPS treatment, LPS + AS did not affect cell adhesion while LPS + LC enhanced cell adhesion (LPS Control 107.14% ± 27.62%; LPS + LC 510.39% ± 115.47%, *p* < 0.01, *n* = 10–12). Cell adhesion during H_2_O_2_ stress was completely abolished as shown in Figure 2d.

### 3.4 Cell migration was significantly affected by high BSA in both cell types and H_2_O_2_ in pMPM, while PC perturbations decreased migration universally

**FIGURE 3:**
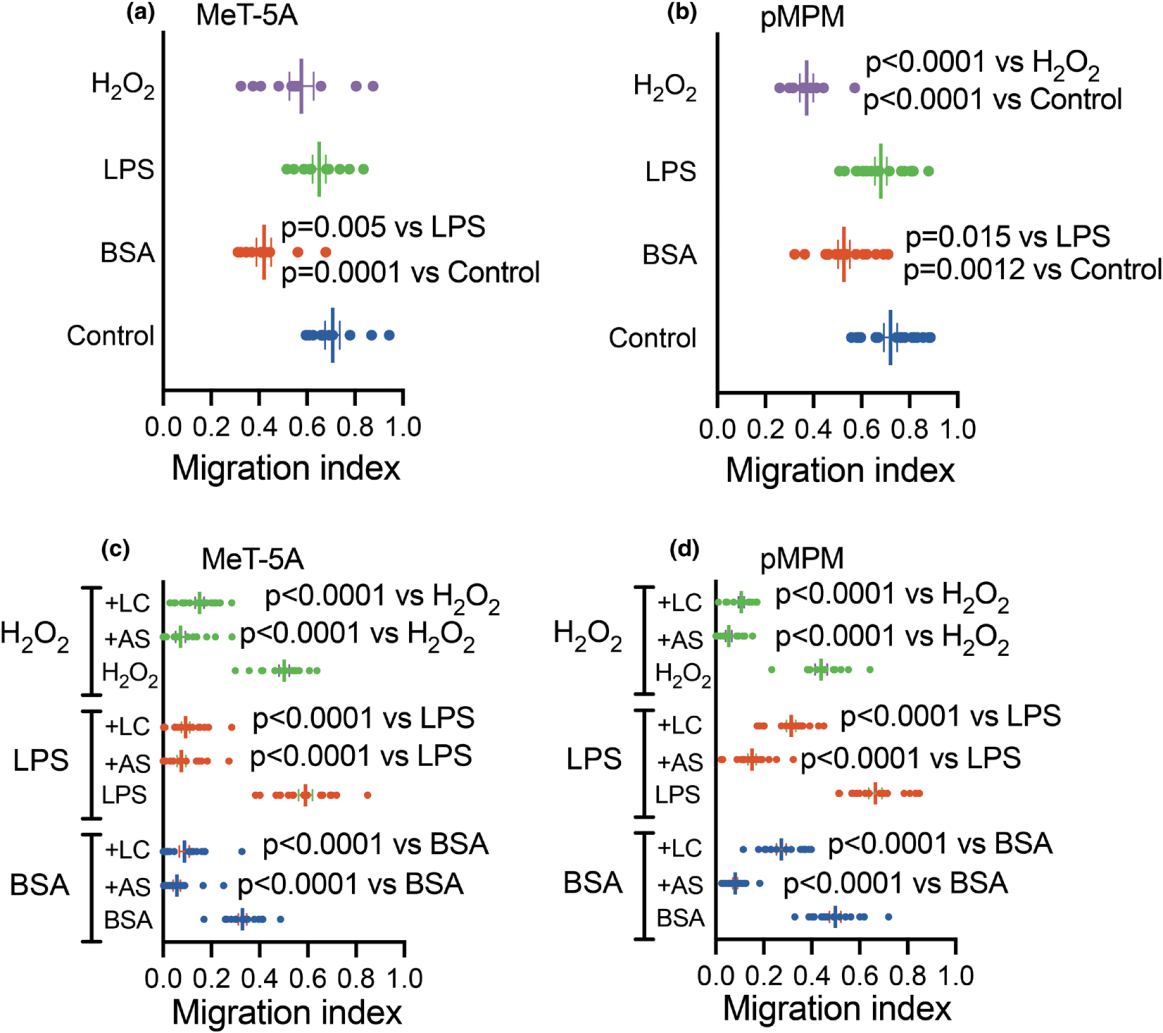
The effect of BSA, LPS, or H_2_O_2_ stimulation during cell migration. Mean MI ± SEM with BSA, LPS, or H_2_O_2_ with treatments (AS, LC) are shown. (a, *n* = 12, 2 experiments; c, *n* = 18, 3 experiments) MeT‐5A, (b, *n* = 18, 3 experiments; d, *n* = 15–18, 3 experiments) pMPM cells. Actual *p*‐values are shown vs. untreated controls or stated for comparisons among the groups.

MeT‐5A and pMPM cells were subject to high BSA, LPS, or H_2_O_2_ extracellular stimulus. 10% FBS‐RPMI was used as a control. In both MeT‐5A and pMPM cells, high BSA hyperosmotic stimulus resulted in a significantly decreased cell migration index as compared to controls (MeT‐5A: Control 0.71 ± 0.03; BSA; 0.42 ± 0.03; *p* < 0.001, *n* = 12; pMPM: Control 0.72 ± 0.03 *n* = 18; BSA; 0.53 ± 0.03; *p* < 0.01, *n* = 18) (Figure 3a,b). In both cases, the MI of the BSA group was also significantly less than the LPS group (*p* < 0.01 for MeT‐5A and *p* < 0.05 for pMPM). In pMPM cells, H_2_O_2_ also resulted in a significant reduction of the MI (Control 0.72 ± 0.03 m = 18; H_2_O_2_ 0.37 ± 0.03 *n* = 10; *p* < 0.001; Figure 3b). The migration index of the BSA group was also significantly decreased compared to the LPS group (*p* < 0.001). Cell migration images from MeT‐5A cell line experiments are provided in the supplemental figures (Figure S1).

Next, we assessed whether the effects of the extracellular stimuli on cell migration could be influenced by perturbation of PC length. In both MeT‐5A and pMPM cells, the addition of a drug that would either cause deciliation (AS) or elongation of the PC (LC) led to universally decreased MI. More specifically, in MeT‐5A cells, the MI was significantly reduced by AS and LC in combination with BSA or LPS or H_2_O_2_ as controls (BSA; 0.329 ± 0.01, BSA + AS; 0.05 ± 0.01, BSA + LC; 0.08 ± 0.02; *p* < 0.001, *n* = 18), (LPS; 0.59 ± 0.02, LPS + AS; 0.07 ± 0.01, LPS + LC; 0.09 ± 0.01; *p* < 0.001, *n* = 18), and (H_2_O_2_; 0.50 ± 0.02, H_2_O_2_ + AS; 0.07 ± 0.02, H_2_O_2_ + LC; 0.15 ± 0.01; *p* < 0.001, *n* = 18) as shown in Figure 3c. Likewise, in pMPM cells (Figure 3d), the mean MI was significantly reduced when PC‐modulating AS or LC was combined with BSA, LPS, or H_2_O_2_, (BSA; 0.49 ± 0.02, BSA + AS; 0.08 ± 0.01, BSA + LC; 0.27 ± 0.02; *p* < 0.001, *n* = 18), (LPS; 0.66 ± 0.02, LPS + AS; 0.15 ± 0.01, LPS + LC; 0.31 ± 0.01; *p* < 0.001, *n* = 18), and (H_2_O_2_; 0.43 ± 0.02, H_2_O_2_ + AS; 0.05 ± 0.01, H_2_O_2_ + LC; 0.10 ± 0.01; *p* < 0.001, *n* = 15). Cell migration images from pMPM cell line experiments are provided in the supplemental figures (Figure S2).

## 4 DISCUSSION

This study investigated PMC responses to extracellular stimuli relevant to pleural effusions, focusing on changes in BBSome gene expression and cell behavior under stress. Using 2D and 3D cultures of MeT‐5A and pMPM cells, we evaluated the gene expression of BBSome components under hyperosmotic, inflammatory, and oxidative stress. BBS expression patterns varied depending on culture conditions, stress type, and cell line, extending previous findings of dimension‐ and malignancy‐dependent BBSome expression changes (Rouka et al., 2023). One striking feature of our findings is that in benign cells, 2D and 3D gene expression patterns are inversed, while in the primary malignant cells the BBSome genes' expression consistently remains significantly down‐regulated or shows a down‐regulation trend. In our view, this may be due to the cells' organization and the nature of the cell type. There is evidence in 2D versus 3D comparative studies in the SiHA cervical cancer cell model. It was demonstrated that inflammatory signals during pathogen‐induced cytokine storm, growth factors, matrix proteins (ECM proteins and associated proteases), angiogenesis, tissue remodeling, migration, and wound healing are differentially regulated (Kumar et al., 2024). Interestingly, in another high‐throughput study on ovarian cancer cells, the choice of ECM scaffold used for 3D tissue organization led to an inverted gene signature compared to 2D cell culture (Kerslake et al., 2023).

While the BBSome expression patterns are fascinating, the nature of cell type and the cell culture model need to be considered. In the context of 3D culture compared to 2D in the HEY ovarian cancer cell line, it has been shown that 3D growth affects a number of processes that can affect tumorigenesis and response to chemotherapies, including the EMT process, multiple cellular stress pathways, DNA integrity pathways, and epigenetic pathways (Paullin et al., 2017).

The proper stoichiometry of BBS components is essential for BBSome function in the primary cilium (PC), and disruptions may impair its role (Mill et al., 2023). There is evidence of redundancy among the BBSome components, especially BBS4 and BBS5. This feature is also demonstrated to be evolutionarily conserved. It was demonstrated that BBS4 and BBS5 regulate the degradative sorting of ciliary sensory receptors (Xu et al., 2015). BBS5 expression is repressed in benign 2D cultures (MeT‐5A) and this appears to be the only exception to the BBSome genes in both benign and malignant mesothelial cells. It can be speculated that under these circumstances there is a likelihood of BBS4 protein substituting BBS5 in ciliary protein complex sorting.

Oxidative stress with H_2_O_2_ altered most *BBS* genes in both MeT‐5A models, with opposing patterns between 2D and 3D, highlighting dimensional effects. This is in contrast to *BBS1* downregulation, which was limited only to the 2D pMPM cultures, while in 3D, none of the BBS gene expression was altered. Previous studies report differing stress responses between benign and malignant mesothelial cells (Katkova et al., 2019; Pellavio et al., 1892). Since PC length and function are sensitive to oxidative stress (Kong et al., 2019; Marion et al., 2011), the findings support PC vulnerability in malignant pleural environments. In addition to the mesothelial setting, in human follicular granulosa cells, the 3D setting confers protection against oxidative stress and thus promotes survival (Zhao et al., 2023). BBS1 in mouse has been demonstrated to have morphological effects on mitochondrial form and function. Loss of BBS1 leads to abnormal mitochondrial morphology and impaired mitochondrial function, as evidenced by reduced oxygen consumption, altered distribution, and changes in calcium flux. Such defects promote mitochondrial hyperfusion, oxidative stress, and altered cellular metabolism. Restoration of normal mitochondrial dynamics can improve these defects, implicating BBSome in regulating oxidative stress via mitochondrial control (Guo et al., 2022). BBS4 and related BBSome proteins may influence cellular stress responses through interactions with the p62‐Keap1‐Nrf2 pathway, a key regulator of antioxidant gene expression. The Nrf2 pathway is activated under oxidative stress to upregulate cytoprotective and detoxifying genes, providing protection against cellular stress. Emerging evidence suggests that modulation of BBSome gene expression can affect Nrf2‐mediated antioxidant responses in certain contexts, including cancer cells (Zhang et al., 2021).

Hyperosmolarity upregulated *BBS1*, *BBS7*, and *BBS18* in 2D MeT‐5A but not in 3D; LPS had no effect. In pMPM cells, all stressors downregulated *BBS* genes in 2D (e.g., *BBS2, BBS4, BBS5, BBS9*), while in 3D only hyperosmolarity decreased *BBS9*. The effect of a hyperosmotic stimulus may involve complex cross‐talk among BBS proteins and end‐effector cell adhesion complexes. In canine kidney epithelial cells, there is demonstrated up‐regulation of the expression of cell adhesion molecule β1 integrin during hyperosmotic stress from NaCl, Urea, and Raffinose (Sheikh‐Hamad et al., 1997). Elsewhere, it has been demonstrated that osmotic stress augments p21 activated kinase (PAK) through the PI3K pathway and, further, that PAK functions downstream, is stabilized by focal adhesions, and in turn stabilizes cytoskeletal re‐arrangement (Chan et al., 2008). BBS8 and BBS9 have been shown to directly interact with Vinculin, that is an integrated part of focal adhesion in mouse renal kidney cells (Hernandez et al., 2013). Acetylated tubulin, the core modification of the primary cilium, is regulated via enzymatic post‐translational modification through the action of alpha‐tubulin acetyltransferase (αTat). In the αTat genomic deleted cells, mouse embryonic fibroblasts show a significantly smaller area of cell‐substratum interaction and less focal adhesion, as assessed by immunocytochemistry (Aguilar et al., 2014). Lastly, functional transcriptomic analyses of mouse retinal pigment epithelial cells demonstrated that BBS8 genomic deletion down‐regulated cell adhesion‐related biological processes (Schneider et al., 2021).

MeT‐5A cells did not show any significant changes during proinflammatory stimulus, irrespective of the dimensional cultures. While LPS affected BBS expression only in 2D pMPM cultures, aligning with its known pro‐tumorigenic role (Liu et al., 2022) and suggesting potential effects in pleural mesothelium, it has mainly been studied in peritoneal contexts (Li et al., 2019; Li et al., 2021). The role of BBSome in inflammatory signaling is not well reported; however, BBS1 and BBS8 have been shown to participate in the T‐cell immune response (Stump et al., 2022) and in brain astrocyte‐mediated inflammatory cytokine signaling, respectively (Singh et al., 2019). In mouse *BBS4* knockout models, it was demonstrated that there was inflammatory infiltration in the renal tissues of *BBS4* deficient mice. This phenotype also had pronounced expression of inducible Nitric oxide synthase, a major, diffusible immune regulator (Zhao & Rahmouni, 2021).

Regarding cell adhesion, hyperosmotic and inflammatory stimuli increased pMPM adhesion but had no effect on MeT‐5A. In addition, PC‐elongating treatment (LC) promoted adhesion in MeT‐5A and pMPM cells upon LPS exposure. Further research is needed to explore the potential implications of these findings. H_2_O_2_ reduced fibronectin adhesion in both. Fibronectin binding via α5β1 integrins is disrupted by oxidative stress, likely through ERK1/2 activation (Zarogiannis, Filippidis, et al., 2013). Hyperosmolarity and inflammation, closely linked (Schwartz et al., 2009), influenced adhesion depending on PC status: elongation increased adhesion, while autotomy reduced it. In terms of migration, MeT‐5A showed slower migration under hyperosmotic conditions, while pMPM migration was inhibited by BSA and H_2_O_2_. Disruption of PC consistently impaired migration, corroborating earlier findings (Jagirdar et al., 2023). These results contrast with reports suggesting that reactive oxygen species promote migration, supporting tissue‐specific H_2_O_2_ roles (Hurd et al., 2012). Given the relevance of PC integrity in cisplatin resistance in MPM (Lee, 2023), our findings underscore the therapeutic potential of targeting PC in pleural malignancy. The M390R mutation in the human *BBS1* gene has a noticeable impact, significantly reducing cell migration and leading to abnormal fibroblast orientation at the edge of the wound. The same mutation in mouse models showed identical loss of migratory speed and direction post wound healing (Guo & Rahmouni, 2019). Studies in mouse *BBS4* and *BBS6* knockout kidney medullary cells have demonstrated that there is a significant reduction in adhesive capability after cell division and lowered cell migration in confluent monolayer cells after wound scratch. It was further demonstrated that the formation of filopodia and lamellipodia extensions is observed in cells presenting a rounded appearance. In the same study, BBS8 knockout in NIH3T3 cells and in the BBS‐null types resulted in marked disorganization of actin fiber assembly (Hernandez et al., 2013).

A limitation of the present study was the lack of direct assessment of PC morphology, frequency and protein expression under the tested conditions. Although changes in BBSome gene expression were observed, transcript abundance does not necessarily reflect alterations in ciliary structure or function. Future studies incorporating immunofluorescence microscopy of ciliary markers (e.g., acetylated α‐tubulin) and quantitative measurements of cilia will be required to establish mechanistic links between BBSome regulation and mesothelial cell behavior. Another important concern is that lithium is a well‐established inhibitor of glycogen synthase kinase‐3β (GSK3β) (Chatterjee & Beaulieu, 2022) and can influence numerous signaling pathways independent of PC. Consequently, some of the observed effects on adhesion and migration may reflect GSK3β‐dependent mechanisms rather than exclusively cilia‐mediated responses. These potential off‐target effects should be considered when interpreting our results. Dose–response analyses were not performed for the drugs tested therefore some effects may partially reflect nonspecific cellular stress or cytotoxicity at the concentrations used. Finally, LPS exposure induces the secretion of cytokines such as TNF‐α, IL‐1β and TGF‐β that are key mediators of inflammation and in the future it would be worthwhile dissecting their exact effects in terms of PC physiology and pathophysiology.

## 5 CONCLUSIONS

We demonstrate that extracellular stimuli relevant to pleural effusions modulate BBSome gene expression and alter mesothelial cell adhesion and migration. These effects depend on cell type and dimensional context. Oxidative stress impaired adhesion and migration across both cell types. In malignant cells, hyperosmolarity and inflammation enhanced adhesion, particularly when PC was elongated. In benign cells, elongation increased adhesion, while autotomy decreased it. Migration was generally reduced by stress, especially under PC disturbance, highlighting the importance of PC homeostasis for mesothelial function and its potential as a therapeutic target.

## AUTHOR CONTRIBUTIONS

**Rajesh M. Jagirdar:** Data curation; formal analysis; investigation; methodology; validation; visualization. **Erasmia Rouka:** Data curation; formal analysis; investigation; methodology; validation. **Eleanna Pitaraki:** Data curation; formal analysis; investigation. **Sotirios I. Sinis:** Data curation; formal analysis; investigation; methodology; resources. **Charalambos Varsamas:** Investigation; resources; validation. **Eleftherios D. Papazoglou:** Investigation; validation. **Lydia Giannakou:** Investigation; methodology. **Panagiotis I. Tzamalas:** Data curation; formal analysis; investigation. **Ourania S. Kotsiou:** Resources; validation. **Anastasios Giannou:** Resources; validation. **Chrissi Hatzoglou:** Methodology; resources. **Najib M. Rahman:** Methodology; validation. **Konstantinos I. Gourgoulianis:** Conceptualization; funding acquisition; project administration; resources; supervision; validation. **Sotirios G. Zarogiannis:** Conceptualization; formal analysis; funding acquisition; investigation; methodology; project administration; resources; supervision; validation.

## FUNDING INFORMATION

The research project was supported by the Hellenic Foundation for Research and Innovation (H.F.R.I.) under the “1st Call for H.F.R.I. Research Projects to support Faculty Members & Researchers and the Procurement of High‐Cost Research Equipment Grant” (Project Number: 3221).

## CONFLICT OF INTEREST STATEMENT

The authors declare no conflict of interest.

## Supporting information


**Figure S1.** The effect of BSA, LPS, H_2_O_2_ without and with PC modulating treatments during cell migration of MeT‐5A cell monolayers. 10% FBS‐RPMI controls; A, B, BSA; C, D, LPS; E, F, H_2_O_2_; G, H. BSA + AS; I, J, BSA + LC; K, L, LPS + AS; M, N, LPS + LC; O, P, H_2_O_2_ + AS; Q, R and H_2_O_2_ + LC; S, T. T0 and T6 indicate the time of image capture. The dotted lines mark the edges of the inflicted wound. The clear area between dotted lines was used to measure the area of the wounded monolayer. For calculating the migration index MI, we divide the difference in areas of T0 and T6 with T0; MI = (Area T0‐Area T6)/Area T0.
**Figure S2:** The effect of BSA, LPS, H_2_O_2_ without and with PC modulating treatments during cell migration of pMPM cell monolayers. 10% FBS‐RPMI controls; A, B, BSA; C, D, LPS; E, F, H_2_O_2_; G, H. BSA + AS; I, J, BSA + LC; K, L, LPS + AS; M, N, LPS + LC; O,P, H_2_O_2_ + AS; Q, R and H_2_O_2_ + LC; S, T. T0 and T6 indicate the time of image capture. The dotted lines mark the edges of the inflicted wound. The clear area between dotted lines was used to measure the area of the wounded monolayer. For calculating the migration index MI, we divide the difference in areas of T0 and T6 with T0; MI = (Area T0‐Area T6)/Area T0.

## Data Availability

Data are available from the corresponding author upon reasonable request.
