# Supplementary material for: Extracellular stressors change BBSome expression of benign mesothelial and primary pleural mesothelioma cells and affect cell adhesion and migration
Source: Physiol Rep. 2026 Jun 16;14(12):e70983. doi: 10.14814/phy2.70983 (PMC13273028; doi:10.14814/phy2.70983)
Supplement: Supplementary file 1 — Figure S1. The effect of BSA, LPS, H2O2 without and with PC modulating treatments during cell migration of MeT‐5A cell monolayers. 10% FBS‐RPMI controls; A, B, BSA; C, D, LPS; E, F, H2O2; G, H. BSA + AS; I, J, BSA + LC; K, L, LPS + AS; M, N, LPS + LC; O, P, H2O2 + AS; Q, R and H2O2 + LC; S, T. T0 and T6 indicate the time of image capture. The dotted lines mark the edges of the inflicted wound. The clear area between dotted lines was used to measure the area of the wounded monolayer. For calculating the migration index MI, we divide the difference in areas of T0 and T6 with T0; MI = (Area T0‐Area T6)/Area T0. Figure S2: The effect of BSA, LPS, H2O2 without and with PC modulating treatments during cell migration of pMPM cell monolayers. 10% FBS‐RPMI controls; A, B, BSA; C, D, LPS; E, F, H2O2; G, H. BSA + AS; I, J, BSA + LC; K, L, LPS + AS; M, N, LPS + LC; O,P, H2O2 + AS; Q, R and H2O2 + LC; S, T. T0 and T6 indicate the time of image capture. The dotted lines mark the edges of the inflicted wound. The clear area between dotted lines was used to measure the area of the wounded monolayer. For calculating the migration index MI, we divide the difference in areas of T0 and T6 with T0; MI = (Area T0‐Area T6)/Area T0. [file PHY2-14-e70983-s001.pdf]

Figure S1

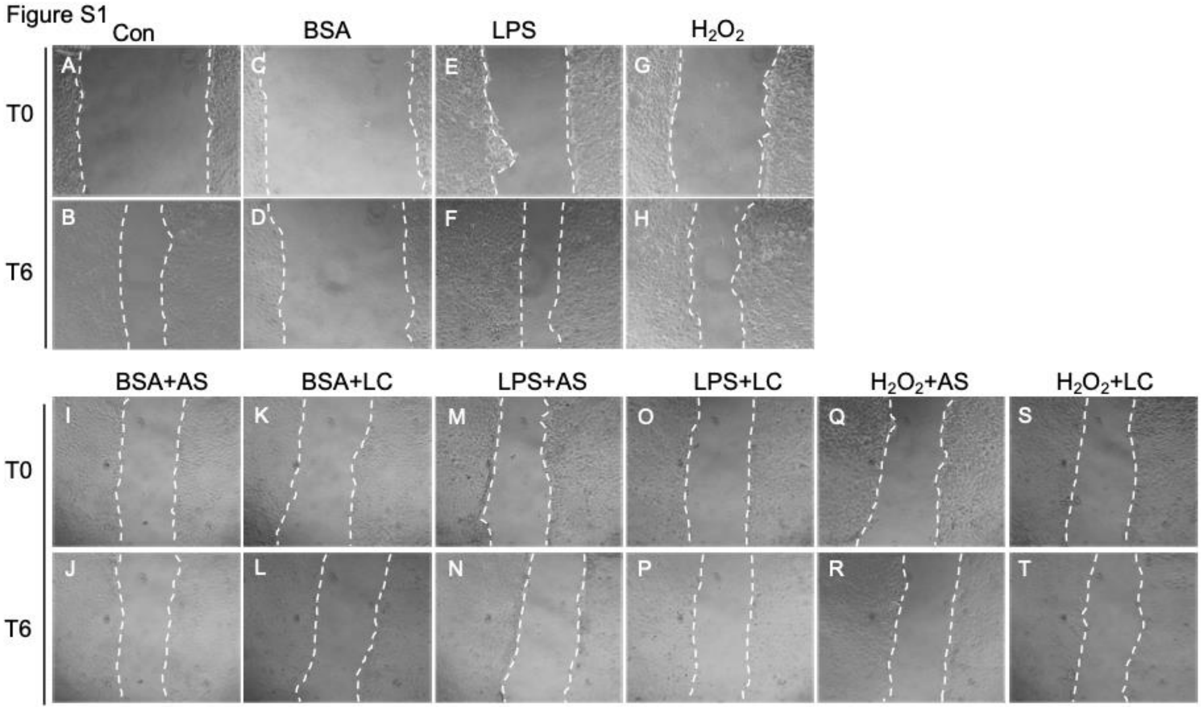

Figure S2

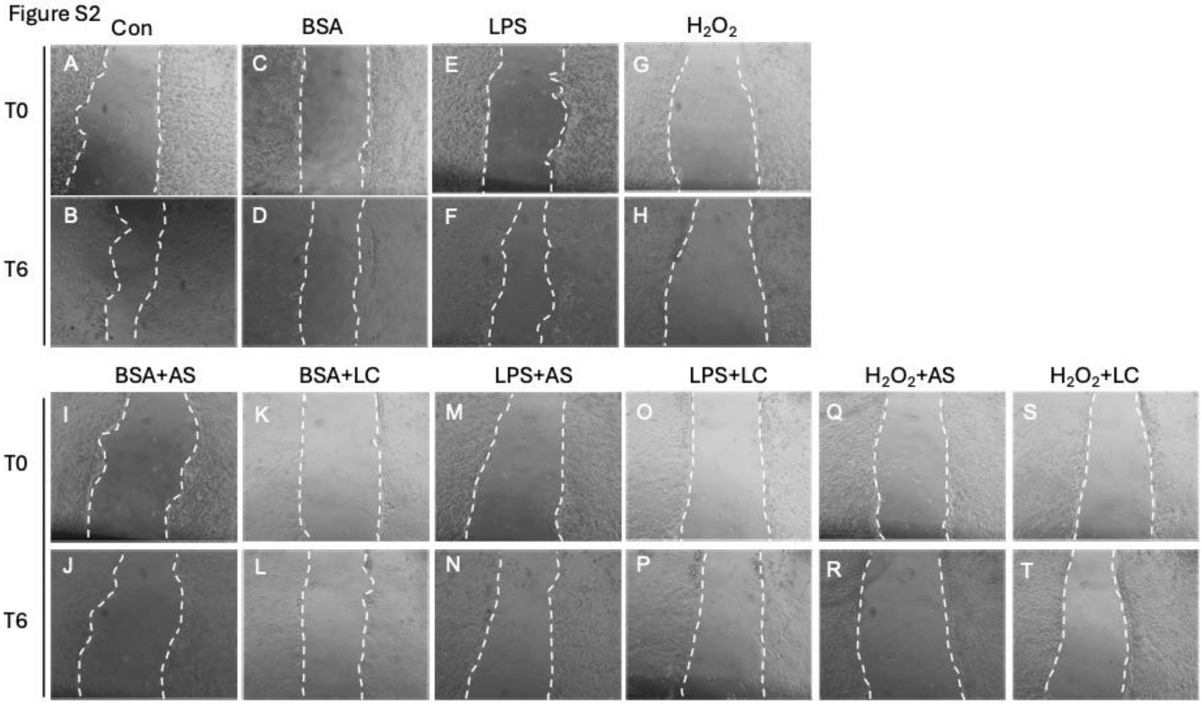

Supplementary figure legend

Figure S1: The effect of BSA, LPS, H_2O_2 without and with PC modulating treatments during cell migration of MeT-5A cell monolayers. 10% FBS-RPMI controls; A,B, BSA; C,D, LPS; E, F, H_2O_2; G,H. BSA+AS; I,J, BSA+LC; K,L, LPS+AS; M,N, LPS+LC; O,P, H_2O_2+AS; Q,R and H_2O_2+LC; S, T. T0 and T6 indicate the time of image capture. The dotted lines mark the edges of the inflicted wound. The clear area between dotted lines was used to measure the area of the wounded monolayer. For calculating the migration index MI, we divide the difference in areas of T0 and T6 with T0; MI = (Area T0-Area T6) / Area T0.

Figure S2: The effect of BSA, LPS, H_2O_2 without and with PC modulating treatments during cell migration of pMPM cell monolayers. 10% FBS-RPMI controls; A,B, BSA; C,D, LPS; E, F, H_2O_2; G,H. BSA+AS; I,J, BSA+LC; K,L, LPS+AS; M,N, LPS+LC; O,P, H_2O_2+AS; Q,R and H_2O_2+LC; S, T. T0 and T6 indicate the time of image capture. The dotted lines mark the edges of the inflicted wound. The clear area between dotted lines was used to measure the area of the wounded monolayer. For calculating the migration index MI, we divide the difference in areas of T0 and T6 with T0; MI = (Area T0-Area T6) / Area T0.
